# Coping Strategy, Social Support, and Psychological Distress Among University Students in Jakarta, Indonesia During the COVID-19 Pandemic

**DOI:** 10.3389/fpsyg.2021.694122

**Published:** 2021-08-26

**Authors:** Zarina Akbar, Maratini Shaliha Aisyawati

**Affiliations:** Department of Psychology, Faculty of Education Psychology, Universitas Negeri Jakarta, Jakarta, Indonesia

**Keywords:** coping strategy, social support, psychological distress, COVID-19, university students

## Abstract

The COVID-19 pandemic has challenged the world for a year, where a study in China showed that the disease increased psychological distress among adolescents and college students, such as anxiety about the academic setback, economic effects, and impact on their daily life. However, a further study examining the impact of the disease on the mental health of students is required. Social support is the most vital psychosocial protective resource, where effective coping can reduce stress levels and prevent individuals from experiencing more severe psychological distress. Therefore, this study investigated the coping strategy, social support, and psychological distress among university students in Jakarta who are also the epicenter of COVID-19 in Indonesia. The psychological distress and coping strategy variable were measured through the Hopkins Symptoms Checklist-25 (HSCL-25) and the COPE Brief instrument, respectively. Meanwhile, the Multidimensional Perceived Social Support-12 instrument was used to measure the social support variable. The study was disseminated via an online form and the number of research subjects included 250 students who matched the research criteria, including DKI Jakarta domicile and active students registered in the area that were confirmed to be COVID-19 positive. According to the results, coping strategies and increased social support were significantly correlated with decreased psychological distress and may serve as the basis for interventions.

## Introduction

Pneumonia Coronavirus Disease 2019, also known as COVID-19 is an inflammatory lung disease caused by Severe Acute Respiratory Syndrome Coronavirus 2 (SARS-CoV-2). Moreover, elderly people and those with a history of disease were known to be more susceptible to the infection due to weak immunity. The World Health Organization (WHO) needed to establish a pandemic status related to COVID-19 due to the continuous increase in the disease cases of more than 118,000 in 114 countries, which originated from Wuhan with the death of 4,291 people (World Health Organization, 2020).

In general, the pandemic is associated with several psychosocial stressors, such as health threats of oneself and loved ones, severe disruption to routines, separation from family and friends, lack of food and medicine, disturbance on economic condition, social isolation due to quarantine or other social distancing programs, and school closings (Shultz et al., 2019). The role of psychological treatment in the patient management process or disaster mitigation schemes in affected communities cannot be ignored (Shultz et al., 2015). Furthermore, in a study conducted in the Chinese region, approximately 35% of the 52,730 respondents affected by the COVID-19 pandemic, experienced psychological distress (Qiu et al., 2020).

Psychological distress in adolescents, can arise due to accumulated anxiety and worry about their health and that of their relatives or friends, changes in sleep and eating patterns, difficulty in thinking or concentrating, worsening chronic health problems, increased alcohol and tobacco use, as well as other drugs (Centers for Disease Control and Prevention, 2020). One global survey discovered that 83% of adolescent respondents agreed that the pandemic worsened pre-existing mental health conditions, mainly due to school closing, loss of routines, and limited social connections (YoungMinds, 2020). The mental health of the students deteriorated due to lose of contact with friends, concerns about the assessment of their grades, and its impact on their university or career prospects. Also, concerns about home learning for practical reasons, stress related to the pandemic, and losing their “safe” place, or non-conducive home environment, contributed to the deterioration (YoungMinds, 2020).

The consideration of psychological distress and mental health symptoms was essential during the COVID-19 pandemic. Also, the unprecedented consequences of the disease, including widespread unemployment and lost income, health-related concerns, and mandatory social isolation are the likely risk factors for increases in forms of psychological distress among the general population. By design, population-based approaches to virus control have imposed significant environmental and contextual constraints for large portions of the population, hence resulting in extensive changes to daily routines and social interactions. Moreover, behavioral theories of psychological distress suggest that reductions in access to environmental or social rewards, and increases in reward-limiting stimuli (i.e., environmental suppressors) predict risk for mental health. By way of constraining daily routines and reducing access to typical sources of social or environmental reinforcement, strict social distancing measures may increase the risk for individuals’ psychological distress (McPhee et al., 2020).

Approximately 25% of the college student samples were reported to experience symptoms of anxiety, which positively correlated with increased concern about the academic setback, economic effects of the pandemic, and impact on daily life (Cao et al., 2020). Therefore, further research examining the impact of COVID-19 on the mental health of students is essential (Grubic et al., 2020). In health psychology studies, social support was associated with reduced cortisol response to stress and better immunity, as well as the most vital psychosocial protective resource according to Taylor (2015). Also, emotionally satisfying social bonds reduce the effects of stress and hopefully, the negative impact possibility of stress on health.

Turner-Cobb et al. (2000) in Taylor (2015) revealed that social support is associated with reduced cortisol response to stress and associated with better immune function or immunity (Herbert and Cohen, 1993). The factor required to suppress the psychological distress experienced by patients, survivors, and their families (Mohammed et al., 2015). Consequently, subjects in a qualitative study stated that the social support they received had a great influence on their ability to face stress (Rabelo et al., 2016). This factor has become fairly important in communities within China during the pandemic because it affects the level of stress and anxiety in medical personnel (Xiao et al., 2020). In another work, social support was negatively correlated with anxiety levels in college students (Cao et al., 2020). However, this study showed that this is a self-protection factor that cannot be ignored.

Regarding the role of social support during a pandemic, people often feel fear and anxiety, not only about the disease but also the uncertainty of its duration, social restriction, and financial problem that arise consequently. Individuals react to psychosocial stresses, such as threats, or actual events (pandemic) in various ways (Taylor, 2019, 2015). However, along with the COVID-19 pandemic, large-scale social restriction and self-isolation can limit the availability and acceptance of social support even though the aim is to stop the spread of the disease. The Indonesian Ministry of Health released guidelines for the public and professionals to maintain mental health as a form of public education related to the importance of social support during the pandemic. Particularly, the social support can be in the form of hotline provision for online psychological services or publication of positive stories about the experiences of people who had been infected with this disease (Indonesian Psychiatric Association, 2020). Coping behavior is a mechanism commonly used in stressful situations regardless of the need for social support as an internal protective factor to overcome distress during a pandemic (Lazarus and Folkman, 1984).

Coping is something that exceeds one’s ability, a cognitive effort, and a constantly changing behavior used to manage external and internal demands considered as a burden (Lazarus and Folkman, 1984). The personality characteristics brought by each person to a stressful event can also affect how they deal with the situation (Taylor, 2015). During a pandemic, everyone can set the best coping mechanism for themselves by regularly seeking information about health risks or responding to the disease by minimizing related information received to avoid anxiety. Taylor (2019) explained that the two responses are the forms of coping executed by the community with their respective advantages and disadvantages. Effective coping can reduce stress levels while preventing individuals from experiencing more severe psychological distress. Therefore, this mechanism is defined as the thoughts and behaviors used for managing internal and external demands from situations that are considered stressful (Folkman and Moskowitz, 2004). Evidently, every individual has different coping steps in dealing with stress.

The Pandemic Management Theory (PMT) is a psychological concept based on the bio-centric health management approach, which includes the definition of six phases of coping with the lockdown burden and the further load process of the COVID-19 pandemic. Bio-centric education teaches the inner basics of how to live as a relationship-oriented and ecological human within a natural and cosmical network. Coping to protect the connection between humans and themselves, others, and the complex of living beings support options (nature) are shown in six bio-centric fields of action during and after pandemics, including (1) maintaining effective communication, (2) maintenance of lively corporeality, (3) contact with one’s own identity and inner oriented self-reflection together with others, (4) construction of life sense and expression of life potentials, (5) expansion of consciousness and perception of the wholeness, and (6) development of ecological awareness and sustainable bio-centric lifestyles and attitudes (Stueck, 2021).

Also, the consideration of protective factors, including social support and coping strategies is essential as a priority in dealing with COVID-19, especially when psychological distress exists in the adolescent and student community. Son et al. (2020) showed that the coping mechanism of students due to stress and anxiety caused by the disease was accomplished by seeking support from others or by helping themselves through adopting negative or positive methods, such as ignoring news about COVID, meditation, breathing exercises, and spiritual approaches. The use of passive coping, continuous exposure to information about the pandemic, and not having a partner as a source of social support, are factors that result in the high level of psychological distress in the Chinese society during the outbreak (Yu et al., 2020).

Reflecting on the psychological evaluation of the pandemic that occurred in May 2020, approximately 69% of the total of 2,364 respondents in Indonesia experienced psychological problems, such as anxiety, depression, and psychological trauma (Indonesian Psychiatric Association, 2020). Further studies are required regarding social support and coping among students in Jakarta, who are the epicenter of COVID-19 in Indonesia. The hypothesis presented by this study is that these factors can reduce the level of psychological distress. Therefore, the results are expected to provide input to policies or learning materials for the community.

## Materials and Methods

### Participants

The purposive sampling technique was used in this study and the number of subjects included 250 students who matched the research criteria, namely Jakarta domicile, those registered in the area, and were confirmed to be COVID-19 positive. Also, the snowball sampling method was used by the investigators to invite a potential study participant group consisting of 10 individuals through the social media platform. Subsequently, the first set of invitees forwarded the invitations to 10 of their contacts whom they considered suitable, and the second set proceeded in the same manner. Participants filled the anonymous basic information online, provided a history of unreported serious mental illness and informed consent, then the participants continued to the three questionnaires.

### Procedures

Eligible participants were asked to complete the questionnaires that contained three distinct sets of items between 20 and 30 min. The first set of items queried demographic characteristics and experience attributed to COVID-19 and included psychological distress questionnaires. Subsequently, participants proceeded to the second set that assessed coping strategy and then the third, which included social support questionnaires. Additionally, two questions appeared at the end of the survey asking the participant to confirm that (1) questions were honestly answered and (2) attention was paid to the survey. Participant data were excluded if they incorrectly responded to > 1 attention checks to control for random responding. The study was disseminated via an online form.

### Measures

This study used the quantitative research method and the variables included psychological distress, coping strategy, and perceived social support.

### Psychological Distress

The Hopkins Symptom Checklist-25 (HSCL-25) explores the symptoms of depression and anxiety and is a validated tool for measuring the level of psychological distress Derogatis et al., 1974. This tool corresponds well to DSM-IV, which defined depression and anxiety disorders, depression, phobia, and somatoform illness using “the Composite International Diagnostic Interview” (CIDI) as a gold standard diagnostic instrument. The 25 items were scored on a scale from 1 (not bothered) to four (extremely bothered) and the “forced” two-factor analyses were in favor of a one-factor solution, although the HSCL-25 measures anxiety and depression dimensions. Therefore, the anxiety, depression, and the mean total HSCL-25 scores were provided in this study, but only the score was used to define psychological distress. This instrument is the result of an adaptation by Turnip and Hauff (2007) with a total of 25 items. Moreover, the reliability coefficient of the HSCL-25 instrument was α = 0.948 and all items of more than 0.3 had a correlation coefficient; hence they are valid from the test results used on 250 participants.

### Coping Strategy

Moreover, the coping strategy variable was measured by the COPE Brief instrument designed by Carver (1997), which consisted of 28 items with two strategy types, namely problem- and emotion-focused coping. Brief COPE consists of 28 items that measure 14 different coping strategies, including active, planning, positive reframing, acceptance, humor, religion, emotional and instrumental support, self-distraction, denial, venting, substance use, behavioral disengagement, and self-blame. The main question was: What do you usually do when you are stressed by a problem? Furthermore, the coping strategies were described in statements, such as “I work or do other things in order not to think about the problem.” Each statement was graded on a four-point Likert scale, where 1 = very seldom, 2 = fairly seldom, 3 = fairly often, 4 = very often. Also, each of the 14 coping strategies was indicated by two items. From the test results used on 250 participants, the reliability coefficient of the COPE Brief instrument was known to be α = 0.822 and only 26 of the 28 items were valid because item numbers 4 and 11 had a correlation coefficient below 0.2 causing them to be invalid.

### Perceived Social Support

The Multidimensional Scale of Perceived Social Support Scale (MSPSS), is a 12-item and self-report instrument, which is easy to administer and was developed by Zimet et al. (1988) to measure social support. This equipment measures individual’s social support from three specific areas with 4 subscales namely family, friends, and significant others. Items were measured on 7- point Likert-type scale from 1 “very strongly disagree” to 7 “very strongly agree.” The MSPSS evaluated perceived social support (PSS) from family (FA), friends (FR), and significant others (SO) as well as quantified the degree to which respondents perceive support from each of these three sources. This instrument has been adapted into Indonesian with reliability α = 0.931.

## Results

A total of 250 participants consisting of university students suspected of having COVID-19, residing in communities where cases of the disease had been reported, 72% female and 28% male, with 58% between ages 20–21 received the invitation to the online survey and finished all the questionnaires. Table 1 shows approximately one-third (38%) of respondents had an academic impact, such as long-distance learning, lectures, internet access, signals, etc., during the COVID-19 pandemic. Of the 250 respondents, 76% had high levels of psychological distress using the Hopkins Symptoms Checklist-25 with a score ≥ 1.75. Table 2 shows the mean scores included problem- 30.77 ± 3.85 and emotion-focused coping 47.94 ± 5.55; friend 20.23 ± 5.28 and family support 19.07 ± 6.52 as well as significant others support 20.22 ± 6.77.

**TABLE 1:** Demographic characteristic of the participants.

Characteristic	Sub-group	N (%)
Gender	Male	70 (28%)
	Female	180 (72%)
Age	18–19	41 (16.4%)
	20–21	145 (58%)
	22–24	64 (25.6%)
The number confirmed positive for COVID-19	No	0
	Yes	250 (100%)
The impact of COVID	Psychological impact (worry, Irritability, sleep difficult, etc.)	165 (30.4%)
	Medial issues (medication, difficulty to participate in clinic or hospital check-up, etc.)	51 (9.4%)
	Academic impact (long-distance learning, lectures, internet access, signals, etc.)	208 (38.4%)

**TABLE 2:** Psychological distress, coping strategies, and social support in Jakarta.

Hopkins symptoms checklist-25	N (%)	
Score < 1.75	60 (24%)	
Score ≥ 1.75	190 (76%)	

**Brief COPE**	**Mean ± SD**	**Range**

Problem–focused coping	30.77 ± 3.85	0–40
Emotion–focused coping	47.94 ± 5.55	0–64

**Multidimensional scale of**		
**perceived social support**	**Mean ± SD**	**Range**

Friends	20.23 ± 5.28	0–28
Family	19.07 ± 6.52	0–28
Significant others	20.22 ± 6.77	0–28

In this study, of the 250 respondents suspected of COVID, 76 and 24% had high and low psychological distress, respectively. The suspected participants had demographic characteristics of mostly female (72%) between the ages of 20–21 years. Table 2 shows these individuals frequently used emotion-focused coping to deal with the stressor, and had support from friends, significant others, and family.

Furthermore, the binary logistic regression also identified that predicted high psychological distress among respondents included emotion-focused coping (*p* < 0.05), support from friend (*p* < 0.05), and significant others (*p* < 0.05). Table 3 shows the following factors, including problem-focused coping and support from family did not predict high psychological distress among student cases. The results showed that there is a negative effect of coping strategies on psychological distress in the individuals affected by the COVID-19 pandemic. In addition, the most positive and negative contribution to psychological distress in this study was emotion- and problem-focused coping, respectively.

**TABLE 3:** Factors predicting high psychological distress in participants.

	95% CI		β	*p*-value
	**Lower**	**Upper**		
Coping style				
Problem focus coping	−0.011	0.039	0.080	0.028
Emotion focus coping	0.019	0.051	0.315	0.000
Social support				
Significant others	−0.028	−0.002	−0.145	0.024
Family	−0.033	0.001	−0.119	0.069
Friend	−0.041	−0.016	−0.265	0.000

## Discussion

### Social Support and Psychological Distress

The study results support other findings which stated there is a negative correlation with the students’ anxiety level in China during the pandemic (Cao et al., 2020). Approximately 24 and 76% of the participants had low and high psychological distress, respectively. This outcome showed that the students’ psychological state was quite worrying because the majority had high psychological distress, which could be caused by impacts in various aspects experienced by these individuals, such as the economic part, students’ academics, and psychology, as well as medical needs, due to the pandemic. Also, having family members, friends, neighbors who suffer from COVID-19 worsened their state during this period. Psychological distress in this study was measured through the suitability of the depression and anxiety symptoms by including some somatic items in participants during the last 7 days. Various forms of concrete or tangible social supports can be provided to students, such as the provision of food or internet networks. This decision was based on the fact that most students’ academics were impacted by the COVID-19 pandemic through long-distance learning, lectures, internet access, signals, etc.

Also, emotional support and information, such as counseling services via telemedicine were required by students because approximately 30.4% of the respondents experienced a psychological impact due to the pandemic. According to Taylor (2015), social support is the most vital psychosocial protective resource, where emotionally satisfying social bonds reduce the effects caused by stress and its bad effects on health. This factor was measured by how it was perceived by students from their friends, family, and significant others. The distress symptoms due to the pandemic were high, including unfounded sudden fear, feeling restless or uneasy, sad, lonely, and less energetic. Moreover, blaming themselves for everything, crying easily, losing appetite, worrying excessively about various things, thinking that everything requires a lot of effort, somatic symptoms, such as headache, and the highest impact of difficulty sleeping. These findings also support another study conducted by Arvidsdotter et al. (2016), which stated that the themes discovered in individuals with psychological distress include difficulties in coping with daily life characterized by a feeling of being haunted by worry and fear, stress, and the inability to calm down. Also, disturbed sleeping by restlessness despite feeling tired, uncertain, and fluctuating emotional state, such as feeling happy, sad, angry, giving up and hopeless, declining tolerance levels, becoming easily frustrated, and irritated with others. Individuals may feel inferior to others, which is indicated by self-depreciation and social isolation and lose one’s grip on life, which is showed by loss of enthusiasm and spirit of life. Furthermore, disorientation and closure of the individuals’ feelings and emotional life result in a lack of empathy and the disappearance of the tendency to give and receive love (Arvidsdotter et al., 2016).

### Coping Strategy and Psychological Distress

The COVID-19 pandemic situation, which is an event of uncertainty, often triggers stress. Lazarus and Folkman (1984) explained that stressful situations cannot be avoided in life, therefore, a coping mechanism is required to overcome them. Also, the study result which showed that coping strategies had a negative effect on psychological distress in students affected by the COVID-19 pandemic, was in accordance with the theory made by Lazarus and Folkman (1984). These individuals stated that coping can help individuals tolerate and master stressful conditions that can trigger psychological distress. The study data also showed that the respondents used problem-focused coping strategies more than the emotion- form. Also, the regression test displayed that the problem-focused coping regression coefficient on psychological distress was negative, hence these strategies are said to be a negative predictor of psychological distress. This is in accordance with a previous study conducted by Mclean et al. (2007), which stated that this mechanism was more frequently used and has a negative effect on student psychological distress. The findings also show that some respondents used emotion-focused coping strategies in dealing with this problem. This is in accordance with the opinion of Lazarus and Folkman (1984), which stated that the majority of the respondents were students with cultural value and personal belief in the formation of personality or cognitive configuration from the culture, hence making them more focused on feelings in dealing with stressful situations. The study result conducted by Vungkhanching et al. (2016) also showed similar evidence, namely the emotion-focused coping strategies affect psychological distress in students.

Also, the results show that several coping strategies have a significant effect on psychological distress, namely the strategy of behavioral disengagement, venting, denial, use of emotional support, humor, and self-blame. The coping strategy that most positively contributes to psychological distress is behavioral disengagement, which involves giving up on making efforts to solve problems. This is in accordance with the opinion of Lazarus and Folkman (1984), which stated that the COVID-19 pandemic situation that occurred in Indonesia can trigger stress and paralyze the anticipatory coping process; hence several individuals choose to conduct behavioral disengagement strategies that ultimately increase their distress level. The strategy that most negatively contributes to psychological distress is the use of emotional support, which is performed by obtaining emotional support or comfort and understanding from others. This was in accordance with the opinion of Lazarus and Folkman (1984), which stated that coping, can come from an external party and consists of the received social support and socio-economic condition. The social support obtained in the form of emotional assistance, information, or real assistance will become the resources for individuals and facilitate them to deal with stressors and reduce distress levels. Also, the results of the descriptive study show that the impact on the respondents’ academics, such as long-distance lectures and difficulty accessing the internet or quotas, and psychology, including stress, anxiety, and irritability had higher category of psychological distress than those with impact on their economic or difficulty in accessing medical care. In addition, the number of confirmed (positive) COVID-19 patients in the area where the respondents live, was directly proportional to the tendency of having a higher psychological distress. This is in accordance with the opinion of Taylor (2019), which stated that several people experience anxiety that weakened them during the pandemic and even interfered with their daily life. Also, depression and sadness are widespread, especially when one or more of closely related persons are positive for COVID-19, where people with high levels of vulnerability and intolerant of uncertainty will be particularly stressed during the pandemic.

According to a study by Moret-Tatay et al. (2016), the Bayesian network model showed higher probabilities of mental health problem symptoms for emotion-focused coping than for the problem-form. However, no differences were discovered regarding gender, hence suggesting the use of problem-focused coping was more recommendable for both male and female university students and may provide some benefits in terms of symptomatic treatments of mental health problems. Based on the results, there was an effect of both strategies on the psychological distress in students affected by the COVID-19 pandemic. According to Carver (1997), coping is an effort used to prevent or reduce threats, losses, or suffering. This process can also be a protective factor because the efforts used to manage excessive stress can reduce psychological distress and have negative consequences on physical health in a short and long period.

### Coping Strategy, Social Support, and Psychological Distress

The study results emphasize the need to investigate coping strategies in the general population and teach them during pandemic outbreaks. This approach may lay a solid foundation for individuals to cope positively and actively with various stress factors and circumstances. The results suggest several considerations for helping the general population in handling the psychological distress caused by the COVID-19 pandemic. These considerations include firstly, fear of COVID-19 is common in the general population worldwide, and the best way to end this occurrence is to learn about the disease and actual risk to others. Secondly, people should be encouraged to work together with colleagues to reduce financial stress, as being unable to work during the pandemic may lead to stress-related job status or financial situation. Thirdly, providing health support, such as a telephone hotline for communication and consulting may help reduce the distress associated with social distancing, quarantine, or isolation. Finally, connecting people with others for giving and receiving social support online can bolster psychological well-being. In addition, feeling lonely and isolated from others is common during the lockdown, and regularly connecting with friends and family through video or phone calls may improve the level of social support.

### Limitations

There are several limitations in this study, which include the surveying process based on network (online) invitation rather than face-to-face random sampling, and requirements of participants to use the Internet. Therefore, it is unclear whether the results can be generalized to individuals who cannot use the Internet. Secondly, respondents’ engagement in the prevention process was not assessed as preventive self-behaviors can also mediate stress levels. Thirdly, the study design was cross-sectional; hence the changes in psychological distress and its predictors throughout the COVID-19 outbreak could not be captured. Therefore, the long-term psychological process and implications of infectious disease outbreaks should not be ignored. Finally, approximately 72% of the respondents were women, which may reduce the generalizability of the findings to the university students in Jakarta population.

## Conclusion

The study results can be used as scientific evidence of the coping strategies effects on psychological distress in students affected by the COVID-19 pandemic. Also, an understanding can be provided on these strategies, psychological distress and their relation to the disease, as well as the enrich results related to coping strategies, and psychological distress. This study can be used as a reference for students in choosing coping strategies as an option in overcoming any pressure that arises due to the COVID-19 pandemic. Psycho-education programs through webinars can be conducted to provide an overview for these individuals on the selection of coping strategies and the right way in dealing with pressure. Therefore, psychological distress was experienced by the students during and after the COVID-19 pandemic.

## Data Availability Statement

The original contributions presented in the study are included in the article/supplementary material, further inquiries can be directed to the corresponding author/s.

## Ethics Statement

The studies involving human participants were reviewed and approved by the Ethics Committee of Faculty of Education Psychology Universitas Negeri Jakarta. The participants in this research also received a complete description of this survey and provided online written informed consent that already reviewed by ethics committee. The patients/participants provided their written informed consent to participate in this study.

## Author Contributions

ZA had full access to all the data, the idea for and designed the study, took responsibility for the integrity of the data, and the accuracy of its analysis. MA did the analysis and edited the manuscript and ZA collected the data while both drafted the manuscript. Both authors agreed to be accountable for all aspects of the work in ensuring that questions related to the accuracy and integrity of any part are appropriately investigated and resolved. Both authors carefully revised the manuscript for important intellectual content and gave final approval for the version to be published.

## Conflict of Interest

The authors declare that the research was conducted in the absence of any commercial or financial relationships that could be construed as a potential conflict of interest.

## Publisher’s Note

All claims expressed in this article are solely those of the authors and do not necessarily represent those of their affiliated organizations, or those of the publisher, the editors and the reviewers. Any product that may be evaluated in this article, or claim that may be made by its manufacturer, is not guaranteed or endorsed by the publisher.
